# Significant difference of differential expression pyroptosis-related genes and their correlations with infiltrated immune cells in sepsis

**DOI:** 10.3389/fcimb.2022.1005392

**Published:** 2022-09-29

**Authors:** Li Wang, Jiting Zhang, Li Zhang, Lingli Hu, Jianhui Tian

**Affiliations:** ^1^ Institute of Antibiotics, Huashan Hospital, Fudan University, Shanghai, China; ^2^ Key Laboratory of Clinical Pharmacology of Antibiotics, National Health and Family Planning Commission, Shanghai, China; ^3^ State Key Laboratory of Genetic Engineering, School of Life Sciences, Fudan University, Shanghai, China; ^4^ Human Phenome Institute, State Key Laboratory of Genetic Engineering, School of Life Sciences, Fudan University, Shanghai, China; ^5^ Huashan Hospital, Fudan University, Shanghai, China; ^6^ Department of Oncology, Shanghai Municipal Hospital of Traditional Chinese Medicine, Shanghai University of Traditional Chinese Medicine, Shanghai, China; ^7^ Department of Integrative Medicine, Huashan Hospital, Fudan University, Shanghai, China

**Keywords:** sepsis, septic shock, pyroptosis, immune cells, diagnosis, integrative analysis

## Abstract

**Background:**

Sepsis is regarded as a life-threatening organ dysfunction syndrome that responds to infection. Pyroptosis, a unique form of programmed cell death, is characterized by inflammatory cytokine secretion. Recently, an increasing number of studies have investigated the relationship between sepsis and pyroptosis. Appropriate pyroptosis can help to control infection during sepsis, but an immoderate one may cause immune disorders. The present study aimed to identify pyroptosis-related gene biomarkers and their relationship with the immune microenvironment using the genome-wide technique.

**Methods:**

The training dataset GSE154918 and the validation dataset GSE185263 were downloaded for bioinformatics analysis. Differentially expressed pyroptosis-related genes (DEPRGs) were identified between sepsis (including septic shock) and healthy samples. Gene Set Enrichment Analysis (GSEA) was performed to explore gene function. CIBERSORT tools were applied to quantify infiltrating immune cells, and the correlation between differentially infiltrating immune cells and DEPRG expression was investigated. Furthermore, based on multivariable Cox regression, the study also utilized a random forest (RF) model to screen biomarkers.

**Results:**

In total, 12 DEPRGs were identified. The expression level of PLCG1 was continuously significantly decreased, while the expression level of NLRC4 was elevated from control to sepsis and then to septic shock. GSEA found that one DEPRG (PLCG1) was involved in the T-cell receptor signaling pathway and that many T cell-related immunologic signature gene sets were enriched. The proportions of plasma cells, T cells CD4 memory activated, and some innate cells in the sepsis group were significantly higher than those in the healthy group, while the proportions of T cells CD8, T cells CD4 memory resting, T cells regulatory (Tregs), and NK cells were lower. Additionally, CASP4 was positively correlated with Neutrophils and negatively correlated with T cells CD4 memory resting and Tregs. Lastly, two biomarkers (CASP4 and PLCG1) were identified, and a nomogram model was constructed for diagnosis with area under the curve (AUC) values of 0.998.

**Conclusion:**

This study identified two potential pyroptosis-related diagnostic genes, CASP4 and PLCG1, and explored the correlation between DEPRGs and the immune microenvironment. Also, our study indicated that some DEPRGs were satisfactorily correlated with several representative immune cells that can regulate pyroptosis.

## Introduction

Sepsis is characterized by a life-threatening, organ dysfunction response to infection and is associated with high mortality and long-term morbidity (Cecconi et al., 2018). Septic shock is described as a subset of sepsis, with circulatory and metabolic abnormalities despite enough fluid resuscitation (Cecconi et al., 2018). Globally, an estimated 48.9 million cases of sepsis and 11.0 million sepsis-related deaths were reported in 2017, leading to serious healthcare costs, especially in Africa and Asia (Rudd et al., 2020). Sepsis patients can manifest a broad spectrum of clinical symptoms, ranging from mild symptoms to the need for ventilation support, organ failure or septic shock, and eventually death (Baghela et al., 2022). During the process of sepsis, the host activates the immune system in response to infection, originating from any infecting organism, which induces the release of proinflammatory and anti-inflammatory mediators and thereby leads to programmed immune cell deaths (Zheng et al., 2021). Once proinflammatory response or immunosuppression becomes excessive, organ dysfunction will occur (Singer et al., 2016).

Pyroptosis, a novel form of programmed cell death, also defined as gasdermin (GSDM)-mediated programmed necrosis, has received much attention due to its association with innate immunity and disease recently (Shi et al., 2017). Caspase-1 (CASP1)-mediated classical and caspase-11/4/5 (CASP11/4/5)-mediated non-classical pyroptosis pathways cleave GSDMs to eliminate intracellular pathogens (Miao et al., 2010; Jorgensen et al., 2017; Zheng et al., 2021). Appropriate pyroptosis can minimize tissue damage and help control infection due to the effective defense against pathogens, but overactivated pyroptosis will trigger severe immunoinflammatory dysfunction and increase the risk of sepsis, septic shock, and secondary infection (Esquerdo et al., 2017; Pu et al., 2017; Gao et al., 2018).

Some studies have investigated the relationship between sepsis and pyroptosis. Intracellular lipopolysaccharide (LPS), a component of Gram-negative bacteria, can activate murine CASP11 (CASP4 and CASP5 in humans) and trigger inflammasome activation, leading to the generation of pore-forming GSDMD-N, which induces cell lysis and subsequent pyroptosis (Shi et al., 2014). GSDMD-N can also activate the Nod-like receptor family pyrin domain containing 3 (NLRP3) sensor-mediated CASP1 activation, resulting in the activation of IL-1β and IL-18 (Meixenberger et al., 2010). NLRP3 inflammasome can be activated by extracellular histones, which could provoke membrane depolarization and enhance oxidative stress (Beltrán-García et al., 2022). In addition, pyroptotic cell death was triggered by GSDMD-N in a phospholipase C gamma 1 (PLCG1)-dependent pattern in macrophages (Kang et al., 2018).

Considering the rapid development of large-scale gene expression profiling, key biomarkers can be screened out for diagnosis or targets. In this study, we investigated the significance of pyroptosis-related gene expression levels in blood between sepsis and healthy individuals, based on the Gene Expression Omnibus (GEO) database. Furthermore, we explored the correlations between pyroptosis and the immune microenvironment.

## Materials and methods

### Data collection and process

The training GSE154918 and validation GSE185263 data files were downloaded from the GEO database annotated by GPL20301 and GPL16791, respectively. The training dataset contains peripheral blood samples from 53 sepsis patients (24 sepsis patients and 29 septic shock patients) and 40 healthy people based on high-throughput sequencing. The downloaded raw reads of the GSE154918 dataset were mapped to transcript and gene level counts by HISAT2.2 (https://ccb.jhu.edu/software/hisat2) and SAMtools (http://htslib.org/), and the bam files were acquired. The htseq2.0 (https://pypi.python.org/pypi/HTSeq) and Cufflinks2.2 (https://anaconda.org/bioconda/cufflinks) were used to perform expression level for mRNAs by calculating FPKM (FPKM = [total_exon_fragments/mapped_reads (millions) × exon_length(kB)]). The validation dataset (GSE185263) contains whole blood RNA-seq data collected from 348 sepsis patients and 44 healthy controls. The expression matrix of the validation dataset was directly downloaded and analyzed.

### Differentially expressed gene analysis and function enrichment analysis

Principal component analysis (PCA) was performed to evaluate intra-group data repeatability in the GSE154918 dataset. Next, to identify key pyroptosis-related genes in sepsis, differentially expressed genes (DEGs) were screened between sepsis (including septic shock) and healthy people, using the “edgeR” package in R (version 4.1.3) (Love et al., 2014). The false discovery rate <0.05 and |log_2_Fold Change| > 1 were set as the cutoff criteria to identify significant DEGs. Volcano maps were drawn using the “ggplot2” package. Gene Ontology (GO) functional and Kyoto Encyclopedia of Genes and Genomes (KEGG) pathway enrichment analyses of DEGs were performed using the online DAVID website (https://david.ncifcrf.gov/home.jsp). *p* < 0.05 was considered as the threshold.

### Differentially expressed pyroptosis-related genes

A total of 33 pyroptosis-related genes (PRGs) were identified by searching previous studies (Shi et al., 2017; Shao et al., 2021; Ye et al., 2021), and they are listed in **Supplementary Table 1**. The abovementioned DEGs intersected with the 33 PRGs to obtain the differentially expressed PRGs (DEPRGs). A protein–protein interaction (PPI) network was constructed using the online STRING website (https://string-db.org), with the interaction score set to 0.4. The expression heatmap of DEPRGs was shown by using “pheatmap” in R.

### Gene set enrichment analysis

Gene Set Enrichment Analysis (GSEA) was also performed based on log_2_Fold Change ranked genes between the healthy and sepsis groups (including septic shock patients) in GSE154918. The background gene sets including “C2, curated gene sets” and “C7, gene immunologic signature gene sets” were obtained from the Molecular Signature Database (MSigDB). A total of 1,000 permutations were used, and an adjusted *p*-value <0.05 was regarded as statistically significant.

### Evaluation of immune cell infiltration

To evaluate the relative proportion of immune infiltrates, CIBERSORT (https://cibersort.stanford.edu/) was used to obtain the infiltrating immune cell matrix (Newman et al., 2015). The 22 types of infiltrating immune cells in each sample were analyzed using package “ggplot2”. A bar graph was drawn to visualize the variance analysis of immune cells between sepsis and healthy samples. Furthermore, Spearman’s correlation analysis was performed on differentially infiltrating immune cells and DEPRG expression.

### Identification of pyroptosis-related biomarkers in sepsis

A random forest (RF) model was established to rank the diagnostic markers for DEPRGs, and the important variables were obtained using MeanDecreaseAccuracy and MeanDecreaseGini. The receiver operating characteristic (ROC) curve was generated to evaluate the model. In addition, DEPRGs were also screened through multivariable Cox regression; *p* < 0.1 was regarded as statistically significant. Finally, according to the importance of the variables, the two most important variables were selected for further analysis. To validate the two biomarkers, an independent external GSE185263 dataset was used. The expression level of the two biomarkers was compared between sepsis and control groups in the GSE185263 dataset.

### Nomogram establishment

The nomogram model was constructed by the “rms” package to predict the occurrence of sepsis. “Total points” was a summary of the score of factors. The performance of the model was evaluated *via* the area under the ROC curve (AUC) analysis.

### Statistical analysis

All statistical analyses were performed with R software (version 4.1.3). A two-sided *p* < 0.05 was considered statistically significant, except for where a certain *p*-value has been noted.

## Results

### Identification of differentially expressed genes

**Figure 1:**
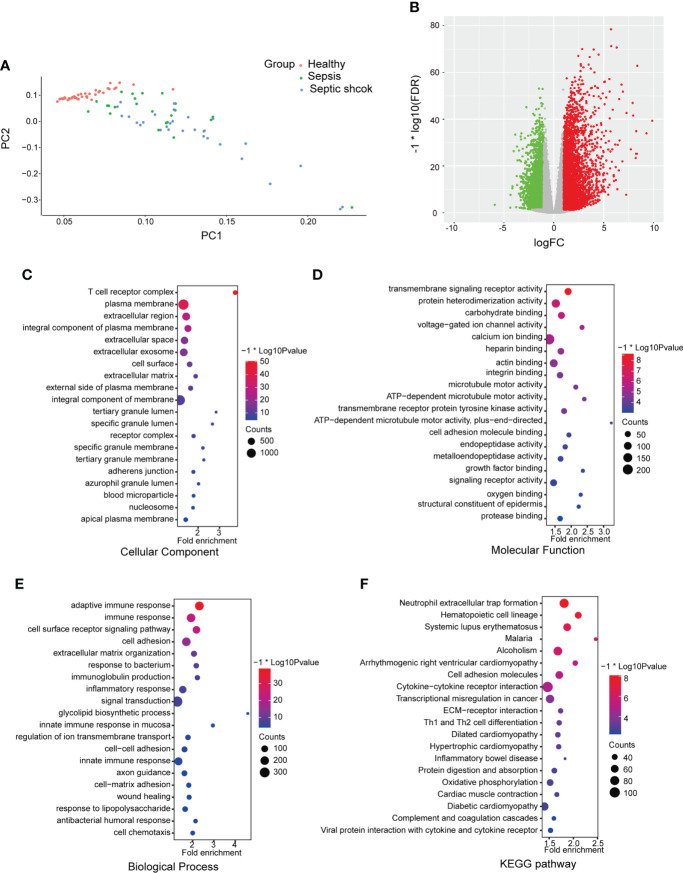
Differential mapping and functional enrichment analysis of sepsis in GSE154918. **(A)** Principal components analyses were performed on all samples in healthy, sepsis, and septic shock groups **(B)** Volcano plots of DEGs between healthy and sepsis (including septic shock). The enrichment analysis shows the top 20 items ranked by *p*-value. **(C)** GO analysis on cellular component (CC), **(D)** molecular function (MF), and **(E)** biological process (BP). **(F)** KEGG pathways. DEGs, differentially expressed genes; GO, Gene Ontology; KEGG, Kyoto Encyclopedia of Genes and Genomes.

The GSE154918 dataset was obtained from the GEO public database. To evaluate each sample’s dissimilarity, PCA showed a good similarity in the intra-group and a different distribution between the sepsis (including septic shock) and healthy groups (**Figure 1A**), which indicated the difference between the two groups. To investigate the two groups, 8,881 DEGs were identified after analyzing the high-throughput data, and the volcano plot is shown (**Figure 1B**). GO and KEGG functional enrichment analyses were performed based on DEGs to investigate the sepsis-related biological function and pathways, and the top 20 terms are shown (**Figure 1**). GO analysis revealed that the cellular component (CC)-associated terms were T-cell receptor complex, plasma membrane, extracellular region, integral component of plasma membrane, and extracellular space (**Figure 1C**). For GO molecular function (MF) analysis, the mainly enriched terms were transmembrane signaling receptor activity, protein heterodimerization activity, carbohydrate binding, voltage-gated ion channel activity, and calcium ion binding (**Figure 1D**). In the biological process (BP), the most enriched annotations were adaptive immune response, immune response, cell surface receptor signaling pathway, cell adhesion, and extracellular matrix organization (**Figure 1E**). The KEGG pathways were neutrophil extracellular trap formation, cell adhesion molecules, cytokine–cytokine receptor interaction, extracellular matrix (ECM)–receptor interaction, and Th1 and Th2 cell differentiation (**Figure 1F**).

### Identification of differentially expressed pyroptosis-related genes

**Figure 2:**
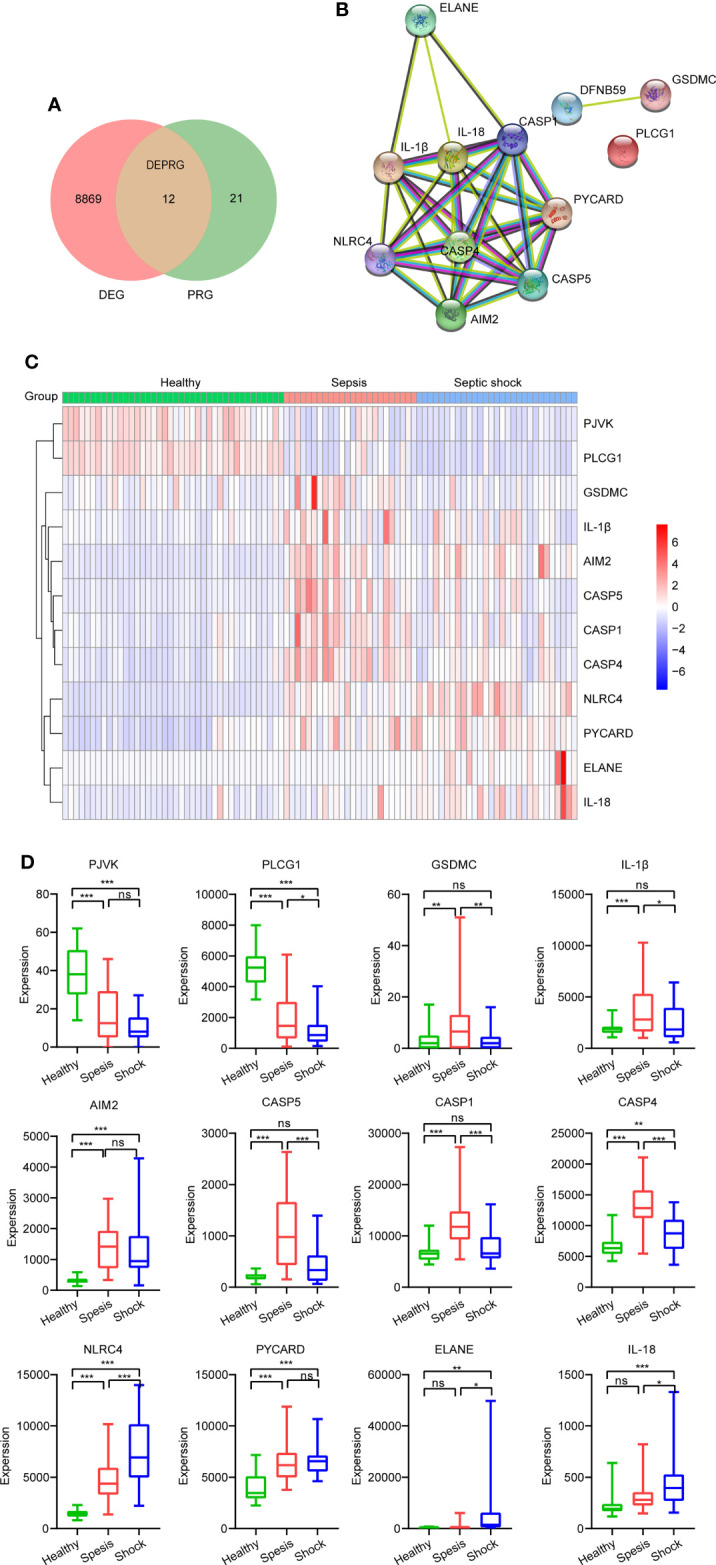
Identification of DEPRGs between healthy and sepsis. **(A)** Venn diagram of DEGs and PRGs in GSE154918. **(B)** The PPI network shows the interactions of the 12 DEPRGs (interaction score = 0.7). **(C)** Heatmap of the 12 DEPRGs’ expression of each sample. **(D)** Boxplot showing expression of each DEPRG in healthy, sepsis, and septic shock patients. *p < 0.05, **p < 0.01, ***p < 0.001. DEPRGs, differentially expressed pyroptosis-related genes; DEGs, differentially expressed genes; PPI, protein–protein interaction. ns is short for no significance

A total of 33 genes were considered as PRGs, and they are listed in **Supplementary Table 1**. According to the Venn diagram (**Figure 2A**), we found 12 PRGs that were differential expressions, including PJVK, PLCG1, GSDMC, IL-1β, AIM2, CASP5, CASP1, CASP4, NLRC4, PYCARD, ELANE, and IL18, and were regarded as DEPRGs. To further investigate underlying relationships between proteins encoded by the 12 DEPRGs, a PPI network is shown in **Figure 2B**. There were 12 nodes and 32 edges. The expression of DEPRGs is shown by a heatmap (**Figure 2C**). In addition, we have shown the expression level of each DEPRG in healthy, sepsis, and septic shock patients by box plots. Compared to that of healthy individuals, the expression level of AIM2, CASP4, NLRC4, and PYCARD was significantly upregulated, while the expression level of PVJK and PLCG1 was significantly downregulated in both sepsis and septic shock patients. Interestingly, the expression level of PLCG1 was continuously significantly decreased during sepsis development, while the expression level of NLRC4 was gradually significantly elevated (**Figure 2D**).

### Gene set enrichment analysis

**Figure 3:**
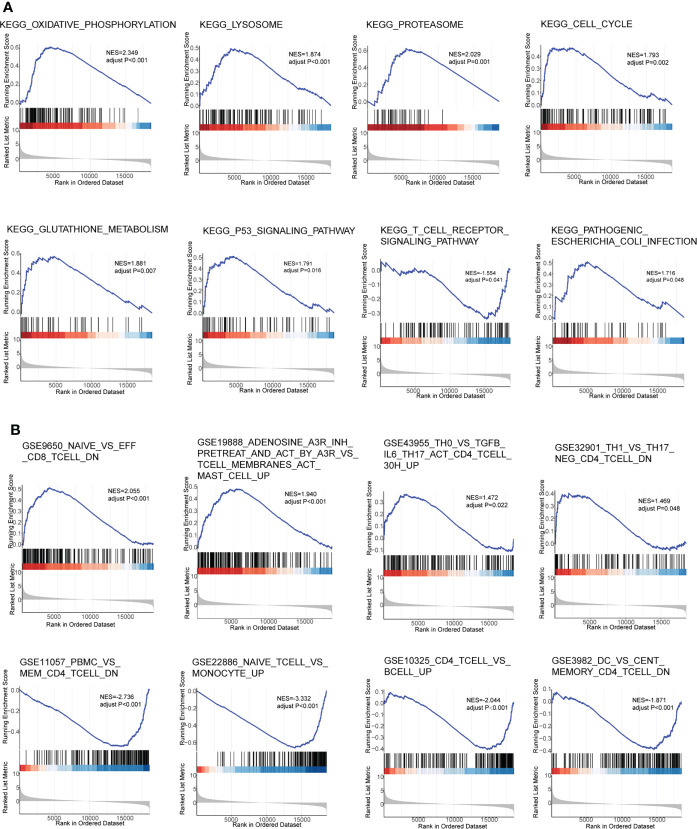
Gene Set Enrichment Analysis in GSE154918. **(A)** KEGG pathways. **(B)** Gene immunologic signature gene sets. KEGG, Kyoto Encyclopedia of Genes and Genomes.

To further explore the possible pathways and gene sets involved in immune functions, GSEA was conducted between sepsis and healthy groups in GSE154918. The results revealed that 18 KEGG pathways were obtained, and many of them were associated with pyroptosis, like KEGG_T_CELL_RECEPTOR_SIGNALING_PATHWAY, KEGG_PATHOGENIC_ESCHERICHIA_COLI_INFECTION, and KEGG_LYSOSOME. Specifically, PLCG1, one of the hub genes, was related to the T-cell receptor signaling pathway, and the normalized enrichment score (NES) was −1.55 in the sepsis group, indicating that this pathway was impaired in sepsis patients. Eight representative KEGG pathways were visualized in **Figure 3A**. Furthermore, 1,426 functional gene sets were enriched in immunologic signature gene sets, and the gene sets related to T cells were significantly enriched, such as GSE9650_NAIVE_VS_EFF_CD8_TCELL_DN, GSE43955_TH0_VS_TGFB_IL6_TH17_ACT_CD4_TCELL_30H_UP, GSE11057_PBMC_VS_MEM_CD4_TCELL_DN, and GSE10325_CD4_TCELL_VS_BCELL_UP. Eight representative plots are shown in **Figure 3B**.

### Immune cell infiltration

**Figure 4:**
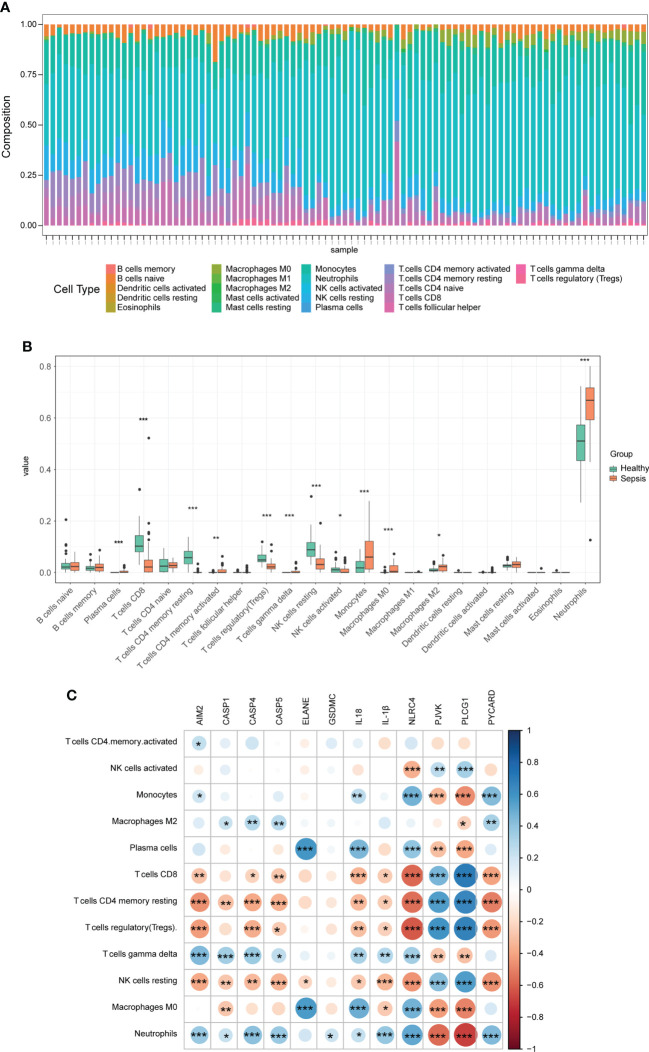
Immune infiltration between sepsis and healthy controls. **(A)** The box-plot diagram demonstrates the composition of immune cells in each sample. **(B)** The difference in immune infiltration between sepsis and healthy controls. **(C)** The correlation heatmap shows the relationship between DEPRGs and immune cells. *p < 0.05, **p < 0.01, ***p < 0.001. DEPRGs, differentially expressed pyroptosis-related genes.

To further understand the immunologic processes, we evaluated the compositions of 22 immune cell types in each sample (**Figure 4A**). Next, we compared the difference in immune cell infiltration between sepsis (including septic shock) and healthy samples, and a box plot is shown in **Figure 4B**. We found that the relative abundance of plasma cells, T cells CD4 memory activated, T cells gamma delta, monocytes, Macrophages M0, Macrophages M2, and Neutrophils in the sepsis group were significantly higher compared with those of normal patients, while the relative abundance of T cells CD8, T cells CD4 memory resting, T cells regulatory (Tregs), NK cells resting, and NK cells activated was lower. We also compared the difference in immune cell infiltration between sepsis and septic shock, and only the proportion of Macrophages M0 and B cells naive had a significant difference, which was significantly higher in the septic shock group than in the sepsis group (**Supplementary Figure 1**). Next, a correlation heatmap was constructed to explore the relationship between DEPRGs and immune cells with significant differences (**Figure 4C** and **Supplementary Table 2**). The graph showed that CASP4 was positively correlated with Neutrophils (r = 0.41) and negatively correlated with T cells CD4 memory resting (r = −0.38) and Tregs (r = −0.37). Furthermore, NLRC4 was positively correlated with Neutrophils (r = 0.52), monocytes (r = 0.48), and Macrophages M0 (r = 0.48), and negatively correlated with Tregs (r = −0.62), T cells CD8 (r = −0.58), and T cells CD4 memory resting (r = −0.55). PLCG1 was positively correlated with T cells CD8 (r = 0.70), Tregs (r = 0.67), and T cells memory resting (r = 0.66) and negatively correlated with Neutrophils (r = −0.68) and monocytes (r=-0.47).

### CASP4 and PLCG1 as pyroptosis-related biomarkers for sepsis diagnosis

**Figure 5:**
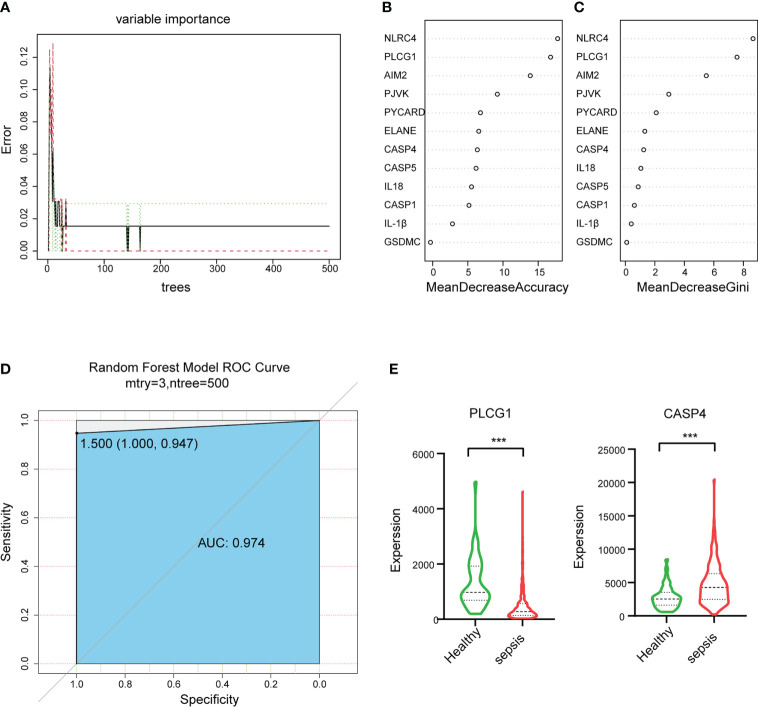
Random forest (RF) algorithm to select hub genes in GSE154918 datasets and validation of hub genes in GSE185263. **(A)** The impact of the number of decision trees on the error rate. **(B)** The importance index of genetic variables, ranked by MeanDecreaseAccuracy and **(C)** MeanDecreaseGini. **(D)** ROC curve. **(E)** The expression level of two biomarkers in GSE185263. ROC, receiver operating characteristic. ***P < 0.001.

**Figure 6:**
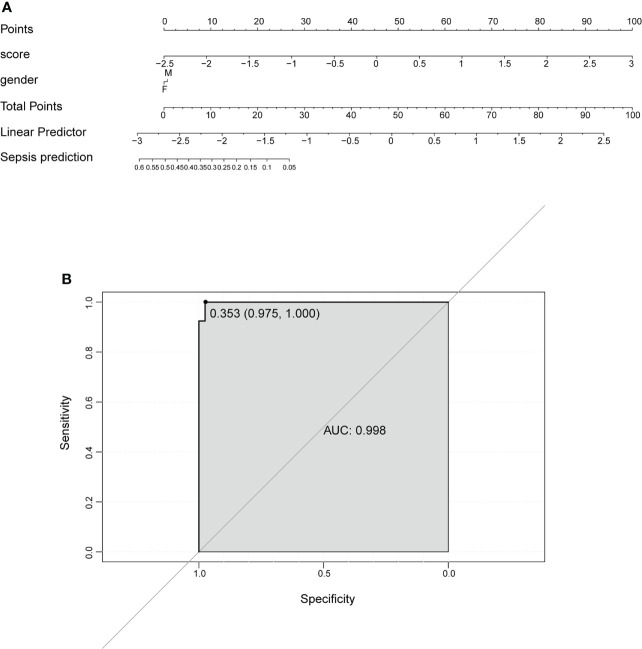
Nomogram to predict the occurrence of sepsis. **(A)** Prognostic nomogram. **(B)** ROC curve to evaluate the performance of the nomogram. ROC, receiver operating characteristic.

To select the hub genes, a random forest was constructed, and the importance of each DEPRG was ranked. The plot shows the relationship between error and the number of decision trees (**Figure 5A**); 500 trees were decided as the number of the final model, which indicated a stable error in this model. The importance of variables was measured based on the output results of MeanDecreaseAccuracy and MeanDecreaseGini (**Figures 5B** **, C**), and NLRC4 and PLCG1 were the most important variables, followed by AIM2, PJVK, PYCARD, ELANE, CASP4, CASP5, and so on. Finally, the AUC was utilized to assess the predictive ability of the model, which was 0.974 (**Figure 5D**). In addition, multivariate Cox regression analysis was performed on the 12 DEPRGs, and only three DEPRGs—PLCG1 (*p* < 0.001), CASP4 (*p* < 0.001), and IL-1β (*p* = 0.09)—exhibited a significant value (*p* < 0.1). However, IL-1β was a less important variable among the 12 DEPRGs according to the RF. Thus, the two genes, PLCG1, and CASP4, were chosen as pyroptosis-related biomarkers for further analysis. In addition, an external GSE185263 dataset was utilized to validate the two genes, and the results showed that the expression of PLCG1 was greatly lower in the sepsis group than in the healthy group, and the expression of CASP4 was higher (**Figure 5E**). The expression of the two biomarkers in the external validation set was in agreement with the training set GSE154918. To verify the accuracy of two genes as biomarkers, a nomogram was established based on the two genes and sex features, using the GS154918 dataset (**Figure 6A**). This nomogram indicated an excellent predictive performance, with AUC values of 0.998 (**Figure 6B**).

## Discussion

Sepsis refers to a syndrome that resulted from a dysregulated host response to infections. Timely recognition and diagnosis of sepsis are always associated with a better prognosis. The pathogenesis of this syndrome is extraordinarily complicated, and it involves many pathophysiological processes, such as unbalanced immunological regulation, inappropriate inflammatory response, autophagy, and pyroptosis (Huang et al., 2019; Zheng et al., 2021). Many previous bioinformatics studies explored sepsis-related cellular and molecular dysregulation mechanisms and identify some biomarkers (Mohammed et al., 2019; Zeng et al., 2021). However, pyroptosis-related mechanisms and biomarkers for sepsis are scarce.

In this study, we first screened DEGs related to sepsis in GSE154918 and conducted an enrichment analysis based on these DEGs. Some enriched terms or pathways were probably associated with pyroptosis, such as the T-cell receptor complex in GO CC, adaptive immune response in GO BP, and neutrophil extracellular trap formation in the KEGG pathway. Next, we identified 12 DEPRGs between healthy and sepsis (including septic shock) samples, demonstrating that DEPRGs do indeed take part in the occurrence and development of sepsis. Our results showed the expression of AIM2, CASP4, NLRC4, and PYCARD was higher and the expression of PVJK and PLCG1 was lower in both sepsis and septic shock patients than in healthy controls. In addition, we found the upregulation of NLRC4 and the downregulation of PLCG1 participated during sepsis progression. Therefore, PLCG1 and NLRC4 may mediate the development of sepsis. It has been reported that suppressing the activation of NLRC4 and lacking AIM2 inflammasomes could decrease IL-1β release and increase the survival rate of septic shock mice induced by LPS (Shin et al., 2017; Xu et al., 2017).

AIM2, an inflammasome sensor, is critical for recognizing both bacterial and viral pathogens and provoking innate immunity (Rathinam et al., 2010). AIM2 can be activated by high-mobility group box 1 (HMGB1) *via* Toll-like receptors (TLR) 2, TLR4, and RAGE/NF-κB pathways in macrophages, and inhibition of AIM2 inflammasome activation can restrain macrophages from releasing IL-1β and IL-18 and protect mice against polymicrobial sepsis and septic death (Xie et al., 2016; Li and Lu, 2020). CASP4 in humans is a homologous protein of CASP11 in mice, which recognizes LPS, mediates GSDMD cleavage and cytokine release, and consequently drives pyroptosis in a non-canonical inflammasome activation manner (Downs et al., 2020). When CASP11 was activated by LPS, pyroptosis and susceptibility to sepsis can be induced by the non-canonical inflammasome through pannexin-1 and the P2X7 signaling pathway, which was downstream of CASP11 (Yang et al., 2015). In order to optimally activate non-canonical inflammasome, type I IFN-α/β and type II IFN-γ were required to upregulate CASP11/4/5, which could also trigger HMGB1 release from innate immune cells effectively (Zhu et al., 2021). HMGB1 plays a pivotal role in the progression of sepsis, which can deliver extracellular LPS into the cytosol of macrophages and endothelial cells to mediate CASP11-dependent pyroptosis and lethality in sepsis (Deng et al., 2018). CASP11-dependent cell death pathway has been reported to aggravate pathologies and shorten survival time in a sepsis mouse model (Napier et al., 2016). NLRC4 exerts a key role in host defense, especially against enteric pathogens (Duncan and Canna, 2018). It mediates the activation of CASP1, which promotes maturation, the release of cytokines, and cell membrane perforation and ultimately induces pyroptosis (Duncan and Canna, 2018). PLCG1 is a kind of membrane-related enzyme and acts as a crucial intermediate process of pyroptosis. It is reported to be associated with cell death and inflammatory response and causes cytotoxicity mediated by GSDMD-N in a calcium-dependent mechanism (Kadamur and Ross, 2013; Kang et al., 2018).

GSEA showed differences in 18 KEGG pathways and 1,426 immunologic signature gene sets between healthy and sepsis groups. The KEGG T-cell receptor signaling pathway was impaired in sepsis patients in this study. Previous studies showed that immunosuppression could occur in sepsis and mediated sepsis-related mortality, and T-cell exhaustion is a serious response (Patil et al., 2016; Jensen et al., 2018). Moreover, the T cell-related gene sets were also enriched based on immunologic signature gene sets, such as NAIVE_VS_EFF_CD8_TCELL_DN, PBMC_VS_MEM_CD4_TCELL_DN, and CD4_TCELL_VS_BCELL_UP.

Sepsis could impair the host’s innate and adaptive immune responses, which are susceptible to primary and secondary infections (Mcbride et al., 2020). Many immune cells participated in this process, such as neutrophils, lymphocytes, and mononuclear/macrophages, which could recognize and devour pathogens and trigger cytokine release to activate other cells (Wen et al., 2022). The results of immune cell infiltration using CIBERSORT algorithm analysis in this study demonstrated that the levels of some innate immune cells, such as monocytes, macrophages M2, neutrophils, and some adaptive immune cells, such as plasma cells and T cells CD4 memory activated, were significantly higher in sepsis patients, while some other adaptive immune cells, such as T cells CD8, T cells CD4 memory resting, and Tregs, were significantly lower. Both monocytes and macrophages are involved in pro- and anti-inflammatory responses in sepsis. Infiltrated monocytes were considered to cause early cytokine storm, which may induce multiorgan dysfunction (MODS) in sepsis. Macrophages were manifested in immunosuppressive responses and played a harmful role in sepsis through CASP11-dependent pyroptosis (Dash et al., 2021; Wen et al., 2022). Neutrophils could direct the cells to the site of infection and create neutrophil extracellular traps (NETs) to enmesh bacteria and trigger local coagulation (Yipp and Kubes, 2013; Nauseef and Borregaard, 2014). However, neutrophils, along with monocytes, could contribute to MODS during sepsis (Dash et al., 2021). Neutrophil pyroptosis is a pro-inflammatory process mediated by GSDMD (Wen et al., 2022). Yang et al. showed that neutrophils could secrete IL-1β through a CASP1-dependent pathway, resulting in a higher mortality rate in mice (Yang et al., 2021). Sepsis could cause immune dysregulation and suppression of adaptive immune cells. CD4+ T cells lose appropriate functions, and CD8+ T cells decrease cytotoxic functions post-sepsis (Nedeva, 2021). Tregs could suppress excessive immune responses and were involved in sepsis-associated immunoparalysis in sepsis patients (Venet et al., 2006; Venet et al., 2009). To understand the relationship between pyroptosis and the immune microenvironment of sepsis, a correlation analysis was conducted. CASP4 was positively correlated with neutrophils and negatively correlated with Tregs. Neutrophils were found sensitive to CASP11-dependent non-classical pathway, and conditional deletion of CASP11 could decrease neutrophil accumulation and pyroptosis (Cheng et al., 2017). A recent study illustrated that the activation of Tregs could attenuate the CASP11-dependent pyroptosis in sepsis-induced lung injury mouse models (Zhang et al., 2021), and depleting Tregs could aggravate lung pyroptosis. This study demonstrated that NLRC4 was positively correlated with neutrophils and Macrophages M0. NLRC4 inflammasomes were present in macrophages and neutrophils, which could release inflammatory mediators *via* CASP1 or CASP8 (Duncan and Canna, 2018). Our study also showed that PLCG1 was positively correlated with T cells CD8 and Tregs and negatively correlated with neutrophils and monocytes. GSEA showed that PLCG1 was related to the T-cell receptor signaling pathway with NES of −1.55 in the sepsis group, indicating that this impaired pathway may mediate the progress of sepsis.

In addition, the present study has good clinical significance. We conducted a diagnosis model including two genes related to pyroptosis, that is, CASP4 and PLCG1, based on the RF model and multivariate Cox regression analysis. Our study revealed that CASP4 and PLCG1 had a good diagnostic value with AUC values of 0.974 in the RF model, and the nomogram further indicated an excellent predictive performance of the two pyroptosis-related biomarkers with AUC values of 0.998. In addition, the two biomarkers were confirmed using an external validation cohort, and the trend of the relative expression was consistent. CASP4 may serve as a potential therapeutic target because previous studies showed that loss of CASP11 could reduce mouse mortality induced by LPS or that deletion of CASP4 could attenuate pyroptosis and cytokine release in human macrophages (Kayagaki et al., 2011; Casson et al., 2015; Downs et al., 2020).

There are also some limitations in this study. The expression level of biomarkers, such as CASP4 and PLCG1, may require further verification using larger clinical samples. The heterogeneous data integration regardless of patients’ age, gender, comorbidities, and other characteristics may contribute to potential, unexpected, and adverse effects on the accuracy of the results of the present study. Therefore, the results should be interpreted carefully.

In summary, we investigated the underlying correlation between pyroptosis and the occurrence of sepsis, and two biomarkers were screened out. Some DEPRGs also had a close connection with the immune microenvironment of sepsis. These findings may offer new insight to investigate the mechanism of sepsis and pyroptosis.

## Data availability statement

Publicly available datasets were analyzed in this study. This data can be found here: https://www.ncbi.nlm.nih.gov/geo/query/acc.cgi (GSE154918; GSE185263).

## Author contributions

LH designed the study and obtained funding. JT participated in the design of the study, reviewed and edited the manuscript. LW and JZ also designed the study, performed the statistical analysis, prepared the figures, and wrote the draft. LZ polished this manuscript. LW and JZ are considered co-first authors. LW, LH and JT are considered co-correspondence authors. All authors contributed to the article and approved the submitted version.

## Funding

This work was funded by the Science and Technology Commission of Shanghai Municipality (Grant No. 22YF1445200).

## Acknowledgments

The authors thank Huisheng Liu (Tongji University) for his technical assistance and manuscript modification.

## Conflict of interest

The authors declare that the research was conducted in the absence of any commercial or financial relationships that could be construed as a potential conflict of interest.

## Publisher’s note

All claims expressed in this article are solely those of the authors and do not necessarily represent those of their affiliated organizations, or those of the publisher, the editors and the reviewers. Any product that may be evaluated in this article, or claim that may be made by its manufacturer, is not guaranteed or endorsed by the publisher.

## Supplementary material

Supplementary Figure 1 The difference in immune infiltration between sepsis and septic shock. *P < 0.05, **P < 0.01, ***P < 0.001.
