# Supplementary figures and images for: Significant difference of differential expression pyroptosis-related genes and their correlations with infiltrated immune cells in sepsis
Source: Front Cell Infect Microbiol. 2022 Sep 29;12:1005392. doi: 10.3389/fcimb.2022.1005392 (PMC9556990; doi:10.3389/fcimb.2022.1005392)

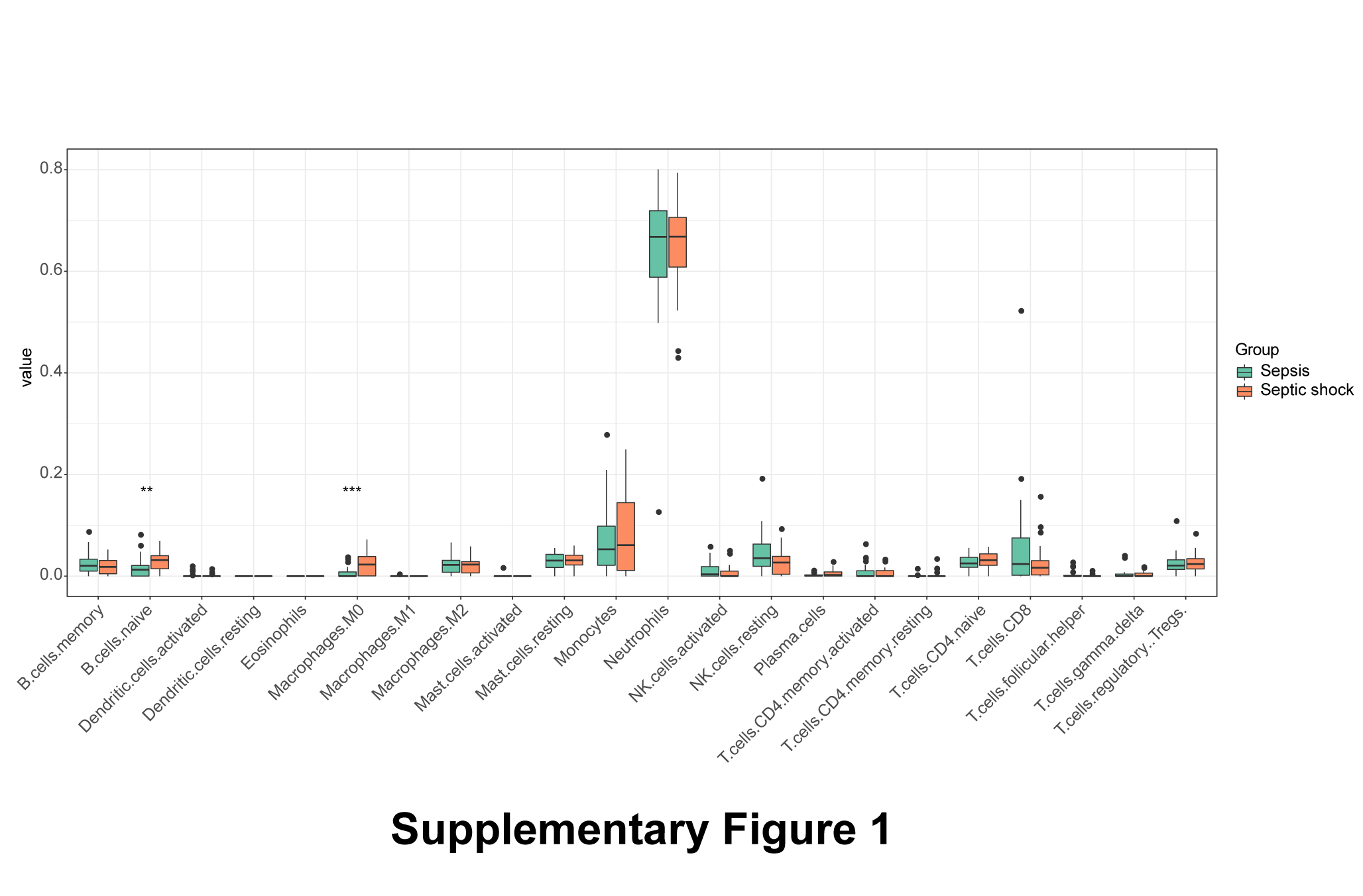

Supplement: Supplementary Figure 1 — The difference in immune infiltration between sepsis and septic shock. *P < 0.05, **P < 0.01, ***P < 0.001. [file Image_1.tif]
